# Yoga practice in England 1997-2008: prevalence, temporal trends, and correlates of participation

**DOI:** 10.1186/1756-0500-7-172

**Published:** 2014-03-24

**Authors:** Ding Ding, Emmanuel Stamatakis

**Affiliations:** 1 Prevention Research Collaboration, Sydney School of Public Health, Level 2, Medical Foundation Building, University of Sydney, 94 Parramatta Rd, Camperdown, NSW 2050, Australia; 2 Physical Activity Research Group (UCL-PARG), Population Health Domain, University College London, London, UK; 3 Department of Epidemiology & Public Health, University College London, London, UK

**Keywords:** Yoga, Exercise, Trends, Prevalence, England

## Abstract

**Background:**

Yoga is a holistic practice that may offer several health benefits. No study has examined the prevalence, temporal trends, or correlates of yoga practice at the population level in a European country and very few such studies exist worldwide. The objective of the study is to examine the prevalence, trends and correlates of yoga practice in England between 1997 and 2008.

**Findings:**

Analysis was conducted in early 2013 using Health Survey for England data. Independent cohorts, representative of adults living in England, were surveyed in annual cycles in 1997-1999, 2003-2004, and 2006/2008. Prevalence of yoga practice (defined as any practice in the past four weeks) was determined at each time point and multiple logistic regression was used to examine temporal trends (using 1997-1999 as reference time point) and the correlates of yoga practice. The prevalence of yoga practice was 0.46% (95% CI: 0.39%-0.52%) in 1997-1999, 0.94% (0.83%-1.06%) in 2003-2004, and 1.11% (0.95%-1.28%) in 2006/2008. Yoga participants in England were more likely to be older, female, degree educated, of non-manual social class, lower BMI, better self-rated general health, inactive occupation, and higher moderate-to-vigorous physical activity. Adjusted for age, sex, social class, and long standing illnesses, there was a significant increasing trend of yoga practice from 1997 to 2008 (2003/04 OR = 1.93, 95% CI: 1.59-2.34; 2006/08 OR = 2.19, 95% CI: 1.77-2.71).

**Conclusions:**

Yoga practice has increased in popularity, though the absolute rates are still relatively low. Future population studies should more comprehensively examine the contexts, settings, styles, correlates and health benefits of yoga practice.

## Background

Yoga is an ancient system of Indian philosophy that emphasises the balance of physical, mental, and spiritual health [1]. As a mind-body practice, yoga includes elements of physical postures, breath control, meditation, and other practice [2]. Yoga has been tested as a modality for stress management [3], pain relief [4], and an intervention to improve the quality of life among people with chronic diseases such as cancer [5,6] and mental health illnesses [7,8].

Although the overall level of intensity is thought to be low [9], a study utilising a human respiratory chamber and heart rate monitors found that certain yoga postures may achieve the recommended level of intensity for cardiovascular fitness [10]. A recent systematic review summarised yoga-based randomised control trials (RCTs) and found that yoga may exceed conventional exercise interventions for improving self-rated health, aerobic fitness, and strength among seniors [11]. Another review of RCTs and other interventions with a pre-post comparison design found that yoga significantly improved the gait, balance, flexibility, lower-body strength, and weight outcomes of seniors [12]. Furthermore, a review of yoga interventions with exercise control groups among adults found that yoga was equal or superior to conventional exercise at improving a series of health outcomes, such as blood glucose, blood lipids, salivary cortisol, and oxidative stress [1]. Although the studies reviewed tended to include small samples, wide variability in interventions, and some had methodological limitations, these studies might provide preliminary evidence for the health benefits of yoga.

Despite the apparent increasing popularity of yoga, no study has examined the prevalence or correlates of yoga practice at the population level in England and very few such studies exist worldwide. The only national data we could identify in the English literature were from the United States, where the estimated prevalence of current yoga practice among adults was 3.7% in 1998 [13] and 5.1% in 2002 [14]. In this study, we used a large population surveillance study to examine the prevalence and trends between 1997 and 2008, and correlates of yoga practice in England.

## Methods

### Sampling and procedures

We used data from the Health Survey for England (HSE), a series of independent annual cohort studies conducted between 1997 and 2008. To improve statistical power for temporal trends analysis we grouped neighbouring years that included physical activity (PA) questions: 1997-1999 (time point 1), 2003-2004 (time point 2), and 2006/2008 (time point 3; no data in 2007). The study sample was recruited using a multistage, stratified, probability design to be representative of adults (≥16 years) living in England. Details on study design and procedures can be found elsewhere [15]. The study has received ethical approval from the Local Research Ethics Councils.

### Measures

Data were collected by interviewers during household visits and questions enquired about socio-demographic characteristics, self-rated health, doctor-diagnosed chronic diseases, long-standing illness/disability/infirmity, depression (General Health Questionnaire) [16], and PA. The interviewers also measured height and weight using standard procedures. The PA questionnaire has been broadly used [17,18] and described elsewhere [19,20]. Yoga practice in the last four weeks was assessed through an open-ended question after collecting information on a fixed list of 10 common activities shown on a card: “Have you done any other sport or exercise not listed on this card?”

### Statistical analyses

We calculated the prevalence of yoga practice at each time point and, using multiple logistic regression and 1997-1999 as the referent point, we computed the odds of participation in 2003-2004 and 2006/08. Time trends analyses were adjusted for age, sex, social class and prevalence of long-standing illness. These covariates were selected based on a previous study on sports trends in England as these demographic variables that have changed over time have acted as confounders in the association between activity prevalence and time [20]. We also used backward stepwise logistic regression to estimate adjusted socio-demographic, personal and health status correlates of yoga participation in the pooled 1997-2008 dataset. Variables tested in the initial model included age, sex, race, social class, educational attainment, occupational physical activity, non-occupational moderate-to-vigorous PA, overweight/obesity (based on body mass index), doctor-diagnosed cardiovascular disease, diabetes, hypertension, depression, and self-rated health.

## Findings

In total, 81,090 respondents had valid data on PA across all time points (n = 38,409 in 1997-1999; n = 27,580 in 2003-2004; and n = 15,101 in 2006/2008). A total of 603 respondents reported yoga practice in the last four weeks (n = 175 in 1997-1999; n = 260 in 2003-2004; n = 168 in 2006/2008). The prevalence of current yoga practice was 0.46% (95% CI: 0.39%-0.52%) in 1997-1999, 0.94% (0.83%-1.06%) in 2003-2004 and 1.11% (0.95%-1.28%) in 2006/2008. Among participants, the mean frequency and total volume of yoga practice was 1.8 ± 1.9 (median/IQR: 1.0/1.3) times/week and 104 ± 218 (median/IQR: 69/60) minutes/week, respectively.

Across the three time points in the unadjusted analyses, yoga participants in England were more likely to be female, of non-manual social class and degree educated (Table 1). There was no significant difference in the age and race (white %) between participants and non-participants. Compared to non-participants, a lower percentage of yoga participants reported having doctor-diagnosed cardiovascular disease, diabetes, hypertension, obesity, and not good self-rated health. There was no difference in the prevalence of depression between participants and non-participants. Yoga participation was linked to lower occupational PA but higher non-occupational overall, moderate-to-vigorous, and vigorous PA. In the adjusted multivariable model using the pooled 1997-2008 dataset, being in older age groups (30-49 and 50+ years of age vs. 16-29 years; OR = 1.56 and 1.96, respectively, *p* < 0.001), being female (OR = 7.41, *p* < 0.001), non-manual social class (OR = 1.70, *p* < 0.001), having a degree (OR = 2.30, *p* < 0.001), being obese (OR = 0.37, *p* < 0.001), having self-reported health (OR = 1.54, *p* = 0.009), inactive occupation (OR = 1.38, *p* = 0.025), and higher non-occupational moderate-to-vigorous PA (OR = 1.01, *p* = 0.003) were correlates of yoga practice. Race, cardiovascular disease, diabetes, hypertension, and depression were not significant in the adjusted model.

**Table 1:** Characteristics of the health survey for England sample by yoga practice status from1997 to 2008

	**Yoga practice**		***p***
	**Yes (n = 603)**	**No (n = 80487)**	
Age (yr), mean (95% CI)	46.7	46.5	0.935
	(45.5, 47.9)	(46.5, 46.8)	
Female (%)	88.4	54.8	<0.001
White (%)	83.3	80.7	0.065
Non-manual social class (%)	76.3	50.5	<0.001
Tertiary degree or higher (%)	32.8	14.9	<0.001
Having physician diagnosed			
Diabetes (%)	2.3	5.4	0.001
Hypertension (%)	14.3	17.9	0.016
Cardiovascular disease (stroke, IHD, angina; %)	2.6	6.4	0.019
Obese (BMI > 30 kg/m^2^; %)	8.8	18.4	<0.001
Self-rated health (fair/bad/very bad; %)	16.4	26.7	<0.001
Depression (%)	14.2	14.9	0.352
Active at work (%)	15.4	26.0	<0.001
Total non-occupational PA (min/week), median (IQR)	300 (416)	147 (343)	<0.001
Moderate-to-vigorous non-occupational PA (min/week), median (IQR)	185 (355)	98 (287)	<0.001
Vigorous non-occupational PA (min/week), median (IQR)	0 (75)	0 (15)	<0.001

Adjusted for age, sex, social class, and long standing illness, there was a significant increasing trend of yoga practice from 1997-1999 to 2006/2008. Adjusted odds for yoga practice in 2003-2004 (OR = 1.93, 95% CI: 1.59, 2.34, *p* < 0.001) and 2006/2008 (OR = 2.19; 95% CI: 1.77, 2.71, *p* < 0.001) were considerably higher than that in 1997-1999.

## Discussion

To our knowledge, this study is the first to examine the prevalence, temporal trends, and correlates of yoga practice among a population representative sample in a European country, while taking into account a plethora of potential PA covariates. Although absolute rates of yoga practice in England were low even in the most recent years, there seems to be an upward trajectory over time.

The prevalence of yoga practice in England was much lower compared to that found in two American studies based on nationally representative samples [13,14]. However, the prevalence rates in the two countries are not comparable due to several reasons. First, the American studies defined current yoga practice as any practice in the last 12 months while our study asked about yoga practice in the last 4 weeks. Second, because in HSE yoga practice was measured based on an open-ended question, respondents were more subject to recall bias and less likely to be prompted to report recent yoga practice. Third, the HSE yoga question was asked in a context of sports and exercise; therefore respondents who practiced less active components of yoga (e.g., breath control, meditation) might not report yoga practice as an exercise. It is reasonable to assume that the measure of yoga in HSE was conservative and likely to capture those who practice an active style of yoga on a regular basis. Compared to other forms of PA in the same HSE cohorts, yoga practice in England was a lot less common than activities such as cycling, running, team sports, and fitness/gym, but more common than martial arts and outdoor water sports [20].

Demographic characteristics of yoga participants in the current study are similar to those in the American studies [13,14] and a German study of internal medicine patients [21]. A typical yoga participant is female, college graduate, and of higher social class. Yoga participants in the current study were also more physically active and less obese. Similar to American yoga participants [13,14], English yoga participants were also more likely to have good or excellent self-rated health compared to non-participants. Compared with the German study which found a higher prevalence of anxiety among patients who practiced yoga, English yoga participants did not seem to differ from non-participants in mental health status as measured by the General Health Questionnaire.

## Conclusions

Yoga practice in England has increased in popularity over the last 15 years, although absolute participation rates are still low. Yoga participants are more likely to be female and tend to broadly have the socio-demographic and health status characteristics of other recreational sports [19]. Despite yoga’s increasing popularity and potential health benefits, it is extremely understudied compared to more conventional physical exercises and therapeutic practices [10]. Given that yoga may benefit human health holistically in ways beyond the traditional western “intensity-focused” exercise paradigm [1], future population studies should capture yoga practice in more detail, including contexts, settings, and styles.

## Competing interests

Ding and Stamatakis have no conflict of interest to report.

## Authors’ contributions

DD and ES conceptualized and wrote the manuscript together. DD conducted literature review and ES conducted data analysis. Both DD and ES read and approved the final draft.

## References

[B1] Ross A Thomas S The health benefits of yoga and exercise: a review of comparison studies J Altern Complement Med 2010 16 1 3 12 10.1089/acm.2009.0044 20105062

[B2] Iyengar BKS Light on Yoga 1976 2 New York: Schocken Books

[B3] Chong CS Tsunaka M Tsang HW Chan EP Cheung WM Effects of yoga on stress management in healthy adults: a systematic review Altern Ther Health Med 2011 17 1 32 38 21614942

[B4] Posadzki P Ernst E Terry R Lee MS Is yoga effective for pain? A systematic review of randomized clinical trials Complement Ther Med 2011 19 5 281 287 10.1016/j.ctim.2011.07.004 21944658

[B5] Cramer H Lange S Klose P Paul A Dobos G Can yoga improve fatigue in breast cancer patients? A systematic review Acta oncologica 2012 51 4 559 560 10.3109/0284186X.2011.637960 22136073

[B6] Levine AS Balk JL Yoga and quality-of-life improvement in patients with breast cancer: a literature review Int J Yoga Therap 2012 22 95 99 23070679

[B7] Uebelacker LA Epstein-Lubow G Gaudiano BA Tremont G Battle CL Miller IW Hatha yoga for depression: critical review of the evidence for efficacy, plausible mechanisms of action, and directions for future research J Psychiatr Pract 2010 16 1 22 33 10.1097/01.pra.0000367775.88388.96 20098228

[B8] Cramer H Lauche R Langhorst J Dobos G Yoga for depression: a systematic review and meta-analysis Depress Anxiety 2013 30 11 1068 1083 10.1002/da.22166 23922209

[B9] Ainsworth BE Haskell WL Whitt MC Irwin ML Swartz AM Strath SJ O’Brien WL Bassett DR Jr Schmitz KH Emplaincourt PO Jacobs DR Jr Leon AS Compendium of physical activities: an update of activity codes and MET intensities Med Sci Sports Exerc 2000 32 9 Suppl S498 S504 10993420 10.1097/00005768-200009001-00009

[B10] Hagins M Moore W Rundle A Does practicing hatha yoga satisfy recommendations for intensity of physical activity which improves and maintains health and cardiovascular fitness? BMC Complement Altern Med 2007 7 40 10.1186/1472-6882-7-40 18053143 PMC2219995

[B11] Patel NK Newstead AH Ferrer RL The effects of yoga on physical functioning and health related quality of life in older adults: a systematic review and meta-analysis J Altern Complement Med 2012 18 10 902 917 10.1089/acm.2011.0473 22909385

[B12] Roland KP Jakobi JM Jones GR Does yoga engender fitness in older adults? A critical review J Aging Phys Act 2011 19 1 62 79 21285476 10.1123/japa.19.1.62

[B13] Saper RB Eisenberg DM Davis RB Culpepper L Phillips RS Prevalence and patterns of adult yoga use in the United States: results of a national survey Altern Ther Health Med 2004 10 2 44 49 15055093

[B14] Birdee GS Legedza AT Saper RB Bertisch SM Eisenberg DM Phillips RS Characteristics of yoga users: results of a national survey J Gen Intern Med 2008 23 10 1653 1658 10.1007/s11606-008-0735-5 18651193 PMC2533368

[B15] Department of Health: Health Survey for Englandhttp://www.esds.ac.uk/government/hse/

[B16] Goldberg DP Gater R Sartorius N Ustun TB Piccinelli M Gureje O Rutter C The validity of two versions of the GHQ in the WHO study of mental illness in general health care Psychol Med 1997 27 1 191 197 10.1017/S0033291796004242 9122299

[B17] Stamatakis E Hamer M Primatesta P Cardiovascular medication, physical activity and mortality: cross-sectional population study with ongoing mortality follow-up Heart 2009 95 6 448 453 10.1136/hrt.2008.152041 18801779

[B18] Williams ED Stamatakis E Chandola T Hamer M Physical activity behaviour and coronary heart disease mortality among South Asian people in the UK: an observational longitudinal study Heart 2011 97 8 655 659 10.1136/hrt.2010.201012 21131381

[B19] Stamatakis E Chaudhury M Temporal trends in adults’ sports participation patterns in England between 1997 and 2006: the Health Survey for England Br J Sports Med 2008 42 11 901 908 18658250 10.1136/bjsm.2008.048082

[B20] Stamatakis E Ekelund U Wareham NJ Temporal trends in physical activity in England: the Health Survey for England 1991 to 2004 Prev Med 2007 45 6 416 423 10.1016/j.ypmed.2006.12.014 17316777

[B21] Cramer H Lauche R Langhorst J Paul A Michalsen A Dobos G Predictors of yoga use among internal medicine patients BMC Complement Altern Med 2013 13 172 10.1186/1472-6882-13-172 23849549 PMC3717114

